# Phalangeal microgeodic syndrome, a case series

**DOI:** 10.1186/1546-0096-9-S1-P28

**Published:** 2011-09-14

**Authors:** J Toelen, T Van Ackere, S Brijs, A Brijs, A Eykens, P Maroteaux, C Wouters

**Affiliations:** 1 Department of Pediatrics, UZ Leuven, Belgium; 2 Department of Radiology, H Hart Roeselare, Belgium; 3 Department of Radiology, St Elisabeth Ziekenhuis Herentals, Belgium; 4 Department of Pediatrics, St Elisabeth Ziekenhuis Herentals, Belgium; 5 Necker-Enfants-Malades Hospital, France

## Background

Phalangeal microgeodic syndrome is a rare bone disorder affecting the fingers of young children, originally described by Maroteaux in 1970.

## Aim

The aim of this case series is to present the clinical and radiological manifestations of three affected pediatric patients, and to illustrate the redundancy of additional investigations if the physician is aware of the disease.

## Patients

The details of three patients with phalangeal microgeodic syndrome are summarized in the table. Three boys presented before the age of 2 years with swelling of the digits of one or both hands. Further clinical examination and laboratory evaluation were normal. On radiological examination multiple small osteolytic areas with sclerotic lining and periostal reactions were visible in the phalanges of the affected hands. All cases were treated with a conservative approach and spontaneous resolution occurred within weeks to months.

## Conclusion

Phalangeal microgeodic is a rare bone disorder presenting with swelling of multiple digits yet alarming radiological signs. These manifestations can be misinterpreted as caused by an infectious, inflammatory or osteolytic condition and prompt clinicians to expand the diagnostic process with MRI or scintigraphic imaging and even biopsy. Timely recognition of clinical and typical radiological signs may prevent unnecessary investigations for this benign condition.

**Table:** 

	Patient 1	Patient 2	Patient 3
**Sex**	male	male	male
**Age**	24m	24m	3m
**Clinical signs**	Painless swelling, redness, both hands	Painless swelling, no redness, left hand	Painful swelling, redness, right hand
**Radiological signs**	Multiple small osteolytic lesions with sclerotic rim and periosteal reaction	Multiple small osteolytic lesions with sclerotic rim and periosteal reaction	Multiple small osteolytic lesions with sclerotic rim and periosteal reaction
**Therapy**	none	none	none
**Resolution**	>3m	3m	1.5m

